# Family Matters: Enhancing Insight in Linked Administrative Data Through Familial Linkage

**DOI:** 10.23889/ijpds.v9i5.2517

**Published:** 2024-09-18

**Authors:** Beverley Phillips, Philip Witowski, Adam Ismail, Windra Sulaiman, Mark Sipthorp, Sharon Williams

**Affiliations:** 1 Centre for Victorian Data Linkage

## Objective

Familial relationships can provide researchers with important insight into genetic, environmental, and social influences across many domains of research. While most administrative datasets do not collect information about relationships, familial linkage is an approach which seeks to identify such relationships among individuals within a linked data environment. We sought to develop a familial linkage resource which permits researchers access to relationship information otherwise not available in unlinked disparate administrative collections.

## Approach

Leveraging birth and marriage registration data, we sought to identify relationships between Victorians. Familial links were formed by summarising relationships that were either explicit in the data (e.g. Parent and Child), or implied (e.g. A parent of a parent is a grandparent). Resulting relationship data was stored in a data asset which interfaces with our linkage infrastructure for easy access and use by linked data end-users.

## Results

Through familial linkage, we have identified over 8.8 million unique relationships for Victorians, spanning 24 relationship subtypes which include both biological and non-biological connections. This data can be linked to all administrative datasets within our linkage environment, however representation varies across sources. Overall, the highest coverage of known relationships is found in datasets which specialise in child services, while older Victorians remain a gap.

## Conclusion

Familial linkage offers new dimensions of insight to researchers than what is accessible in source data alone. This information enables our data end-users to gain critical insights into the complex interplay between biological and social influences on Victorians’ health and well-being.

